# Post Approval Human Papillomavirus Vaccine Uptake Is Higher in Minorities Compared to Whites in Girls Presenting for Well-Child Care

**DOI:** 10.3390/vaccines1030250

**Published:** 2013-07-17

**Authors:** Jennifer Young Pierce, Jeffrey E. Korte, Laura A. Carr, Catherine B. Gasper, Susan C. Modesitt

**Affiliations:** 1 Division of Gynecologic Oncology, Medical University of South Carolina, 96 Jonathan Lucas Street, CSB 634, MSC 619, Charleston, SC 29425, USA; E-Mail: cbgasper@gmail.com; 2 Department of Public Health Sciences, Medical University of South Carolina, 135 Cannon St., Charleston, SC 29425, USA; E-Mail: korte@musc.edu; 3 Department of Pediatrics, Medical Center Boulevard, Wake Forest Baptist Medical Center, Winston-Salem, NC 27157, USA; E-Mail: lac6e@virginia.edu; 4 Department of Obstetrics & Gynecology, University of Virginia Health System, Charlottesville, VA 22903, USA; E-Mail: scm6h@hscmail.mcc.virginia.edu

**Keywords:** human papillomavirus, HPV, vaccine, barriers, adolescent, well child care, cervical cancer prevention

## Abstract

Since introduction of the human papillomavirus (HPV) vaccine, there remains low uptake compared to other adolescent vaccines. There is limited information postapproval about parental attitudes and barriers when presenting for routine care. This study evaluates HPV vaccine uptake and assesses demographics and attitudes correlating with vaccination for girls aged 11–12 years. A prospective cohort study was performed utilizing the University of Virginia (UVA) Clinical Data Repository (CDR). The CDR was used to identify girls aged 11–12 presenting to any UVA practice for a well-child visit between May 2008 and April 2009. Billing data were searched to determine rates of HPV vaccine uptake. The parents of all identified girls were contacted four to seven months after the visit to complete a telephone questionnaire including insurance information, child’s vaccination status, HPV vaccine attitudes, and demographics. Five hundred and fifty girls were identified, 48.2% of whom received at least one HPV vaccine dose. White race and private insurance were negatively associated with HPV vaccine initiation (RR 0.72, 95% CI 0.61–0.85 and RR 0.85, 95% CI 0.72–1.01, respectively). In the follow-up questionnaire, 242 interviews were conducted and included in the final cohort. In the sample, 183 (75.6%) parents reported white race, 38 (15.7%) black race, and 27 (11.2%) reported other race. Overall 85% of parents understood that the HPV vaccine was recommended and 58.9% of parents believed the HPV vaccine was safe. In multivariate logistic regression, patients of black and other minority races were 4.9 and 4.2 times more likely to receive the HPV vaccine compared to their white counterparts. Safety concerns were the strongest barrier to vaccination. To conclude, HPV vaccine uptake was higher among minority girls and girls with public insurance in this cohort.

## 1. Introduction

Human papillomavirus (HPV) is the most common sexually transmitted disease in the United States and is associated with 99% of all cervical cancers [1,2]. In the United States in 2012, approximately 12,170 women were expected to be diagnosed with cervical cancer, and 4,220 died of their disease [3]. Infection with HPV starts at a young age with estimates of cervical HPV prevalence in women aged 18–25 ranging from 27% to 60% [4,5]. Racial disparities result in differential rates of cervical cancer and mortality from cervical cancer. In Virginia, black women have a higher risk of cervical cancer than white women and are twice as likely to die of the disease [6].

A vaccine to protect against human papillomavirus was first introduced in 2006 in the United States (Gardasil®, Merck & Co, Inc., Whitehouse Station, NJ, USA). This quadrivalent HPV vaccine protects against viral types 6, 11, 16, and 18, which are known to cause approximately 90% of genital warts and 70% of cervical cancer [7,8]. Cervarix® was FDA approved in 2009 and protects against HPV 16 and 18 (GlaxoSmithKline, Brentford, UK). In March 2007 the Center for Disease Control (CDC) recommended that all girls aged 11–12 years old receive the HPV vaccine as part of their routine adolescent vaccinations [9]. Other vaccines recommended for this adolescent age group include the meningococcal, tetanus, diphtheria and pertussis booster (Tdap), and varicella (if the child has not been infected with chickenpox).

It is imperative that the HPV vaccine reach all populations in order to significantly impact rates of cervical cancer. The National Immunization Survey for Adolescents (NIS-Teen) documents adolescent immunization for ages 13–17. These data show national HPV vaccination rates fall below recommended levels and are significantly lower than other adolescent vaccinations [10]. Previous studies have demonstrated that parental decisions to have their children vaccinated are associated with knowledge about HPV, physician recommendation of the vaccine, and belief in the safety and efficacy of the HPV vaccine [11]. A recent in depth analysis of the NIS 2008 regarding HPV vaccination, the first year of HPV vaccination reporting, demonstrated differential rates of vaccination completion by race with minority girls less likely to complete the series [12]. Further, well child visits, insurance status, and provider recommendation were noted to be associated with vaccination [13]. The most recent NIS-Teen data shows differential rates of HPV vaccine uptake by race and socioeconomic status with minority races and low SES having statistically higher rates of HPV vaccine initiation [10]. However, further analysis is still pending regarding the reason behind these trends. In Virginia, doctors reported that cost and reimbursement were the most frequently encountered barriers preventing vaccination [14].

In March 2007, the Virginia governor signed into law House Bill 2035 (identical to Senate Bill 1230) requiring the HPV vaccine for girls on or after their 11th birthday but allowing parents to exempt their child via a verbal opt out effective 1 October 2008 [15]. Given that the mandate did not go into effect until October 2008, it did not change school admission requirements until the academic year beginning August 2009. This is the only school mandate in the country and it remains to be seen if it will achieve higher rates of vaccine uptake and decreased racial differences seen with other vaccine mandates [16,17].

This study was conducted to determine whether insurance status and other demographic factors play a role in HPV vaccine uptake and completion among girls presenting for a well-child visit at age 11–12 years old. This study also documents baseline vaccination rates for the year prior to enactment of the Virginia school mandate.

## 2. Experimental Section

### 2.1. Cohort

This prospective cohort was developed by searching the University of Virginia Clinical Data Repository (CDR) at 3 month intervals for all girls aged 11–12 years presenting for a well-child visit between May 2008–April 2009. These dates were chosen to capture the vaccination uptake for the school year prior to enactment of the Virginia HPV vaccine mandate. This included all patients seen at UVA-supported family medicine practices and pediatric practices in a 50 mile radius including urban, suburban, and rural populations. Clinical and demographic data were also collected, including age, race, insurance status, and vaccination status for HPV, Meningitis, Tdap, and Varicella. The primary outcome was HPV vaccine uptake as defined by receipt of ≥1 dose confirmed by billing data. Final collection of HPV vaccine uptake and number of injections received was collected six months after the last identified well-child visit. Of note, this study only evaluated girls as the HPV vaccine had not yet been approved for use in boys at the time of data collection.

To provide greater in depth analysis of these findings, the parents of these girls were then contacted 4–7 months after the initial well child visit to allow for time to complete the HPV vaccination series in three batched samples. A cohort of parents/guardians of the original population agreed to participate in the telephone questionnaire.

### 2.2. Questionnaire Design

A 50-item telephone questionnaire was developed that took the parents approximately 10 min to complete. The questionnaire included validated questions on insurance status and demographics, previously studied questions on HPV vaccination attitudes and behaviors designed utilizing constructs from the Health Belief Model [18], as well as additional questions regarding other vaccination status, relationship and trust in the provider, and usual health information sources. Lastly, previously studied HPV knowledge questions were utilized. The questionnaire was piloted on a subset of parents who were not included in the final data analysis. All questions referred specifically to the quadrivalent vaccine as the bivalent vaccine was not available in these locations.

Parents were contacted first with an advance letter and either called in to the survey center or verbally consented to participate over the telephone. The University of Virginia Center for Survey Research (CSR) trained all interviewers and conducted all interviews and all answers were entered to a database for later data analysis. The parent or guardian who said they were the most familiar with the child’s health history was asked to participate in the survey. To reduce non-response bias CSR made several efforts at “conversion calling” for households where a potentially eligible respondent had refused to participate once or twice.

### 2.3. Statistical Analysis

Data analysis included frequency distribution of race, insurance status, and vaccination status in the original population and the cohort participating in the telephone questionnaire using SPSS version 18.0 (SPSS, Inc., Chicago, IL, USA) and SAS, version 9.1 (SAS, Cary, NC, USA). The primary outcome of HPV uptake ≥1 dose received was converted to a binary variable. Associations were determined by chi-square test for categorical variables with *p* < 0.05 considered statistically significant. Relative risk and 95% confidence intervals were calculated for all cohort data in 2 × 2 tables. Likert scales to assess vaccination attitudes were dichotomized for agree or disagree with the attitude. After preliminary analysis, demographic, knowledge, and attitude data from the telephone questionnaire were utilized in multivariable logistic regression. All factors significantly associated with HPV vaccine uptake or refusal (*p* < 0.1) were retained in the model. The final model was fit with backwards stepwise elimination of nonsignificant variables. Sensitivity analysis was conducted to verify the relationship between the variable and the outcome of HPV vaccine uptake.

### 2.4. Power Analysis

Based on demographics of the pediatric population at UVA in 2008, 60% of patients had private insurance. An HPV vaccination rate of 40% based on the National Immunization Survey from 2008 was used as a baseline assumption [19]. In order to detect a difference in vaccination rates of 15% among privately insured patients compared to publically insured or self-pay patients with 80% power, a sample size of 370 patients was calculated.

## 3. Results and Discussion

### 3.1. Results

Five hundred and fifty 11–12 year old female patients were identified in the total population of girls seen for a well-child visit between May 2008–April 2009. Of these patients, 72.5% were nonhispanic white race, 53% had private insurance, and 48.2% were vaccinated against HPV during the study period (Table 1).

**Table 1:** Demographics of total population compared to cohort of telephone interview respondents.

		Population		Cohort		*p*-value
		n = 550		n = 242		
**Age**	**11**	350	63.6%	152	62.8%	0.990
	**12**	200	36.4%	87	36.0%	
**Race**	**Nonhispanic white**	396	72.0%	183	75.6%	0.981
	**Black**	115	20.9%	38	15.7%	
	**Other**	39	7.1%	27	11.2%	
**Insurance**	**Private**	292	53.1%	201	83.1%	0.944
	**Public**	175	31.8%	38	15.7%	
	**Self-pay**	22	4.0%	3	1.2%	
	**Unknown**	61	11.1%	0	0.0%	
**HPV vaccination**	**0**	284	51.6%	122	50.4%	0.954
	**≥1**	265	48.2%	106	43.8%	
	**≥3**	128	23.3%	42	17.4%	
	**Unknown**	0	0.0%	14	5.8%	

In this population, there were noted to be significant associations of HPV vaccination with insurance status and race. Girls with private insurance were less likely to receive the HPV vaccine compared to girls with public insurance or self-pay. Conversely, girls with public insurance were 36% more likely to be vaccinated as compared to girls with private insurance (Table 2).

**Table 2:** Human papillomavirus (HPV) vaccine uptake (≥1 dose received) by insurance and race in population of 11–12 year old girls presenting for well child visits between May 2008–April 2009.

		HPV vaccine uptake	%	RR	95% CI
**Insurance status**	**Private **	130/292	44.5%	0.85 *	0.72–1.01
	**Public **	103/175	58.9%	1.36 ^†^	1.15–1.61
	**Self-pay ^§^**	11/22	50.0%		
	**Unknown ^§^**	21/61	34.4%		
**Race**	**Nonhispanic white **	172/396	43.4%	0.72	0.61–0.85
	**Black **	70/115	60.9%	1.36 **	1.13–1.63
	**Other ^§^**	23/39	59.0%		
**Total**		265/550	48.2%		

* Compared to publically insured or self-paying patients, Patients with unknown insurance excluded given heterogeneity of the group. ^†^ Compared to privately insured patients. ^§^ Statistical comparisons not performed given small numbers in groups. Compared to black or other race. ** Compared to nonhispanic white race.

Further, nonhispanic white girls presenting for well child visit were overall almost 30% less likely to initiate vaccination compared to all minority girls (RR 0.72, 95% CI 0.61–0.85). Compared to nonhispanic whites, black girls were 36% more likely to have received at least 1 dose of the HPV vaccine. There were no significant differences in HPV vaccination completion rates by race among those who received at least one dose with 84/172 (49%, 95% CI 41%–56%) of nonhispanic whites and 71/154 (46%, 95% CI 38%–54%) of minority girls completing the three injection series.

**Figure 1:**
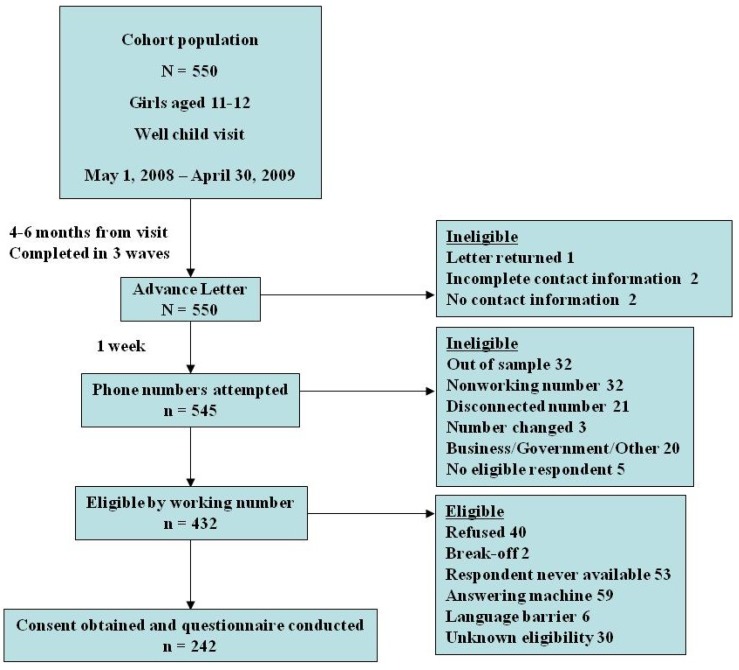
Diagram of cohort originating from study population.

All 550 families from the original population were considered for participation in the telephone questionnaire (Figure 1).

Four hundred and thirty-two households with a working number were approached for participation in the questionnaire and 242 parents or guardians agreed to participate in the telephone interview for a response rate of 56%. (Of note, 219 of the 242 participants were parents. Thus, the survey respondents are referred to as parents even though 23 individuals were guardians of other familial description.) See Table 1 for demographic comparisons of the cohort participating in the telephone questionnaire compared to the entire population.

All parents confirmed the child had a recent visit to the doctor. Eighty-nine percent of participants went with the child to the documented visit, of whom 79% remember the doctor discussing the HPV vaccine. The majority of respondents confirmed that the child had been seeing this physician for 5 years or greater.

Overall, 92% of parents were aware of the HPV vaccine, and 85% reported that the HPV vaccine was recommended for their child. In regards to HPV vaccine knowledge, 69% of respondents agreed that the vaccine protects against cervical cancer, but only 20% also identified that the quadrivalent vaccine protects against genital warts. The majority of respondents (59%) agreed that the HPV vaccine is safe. Twenty percent of parents felt that their child did not need the HPV vaccine. There were no statistically significant differences in these attitudes compared by race.

**Figure 2:**
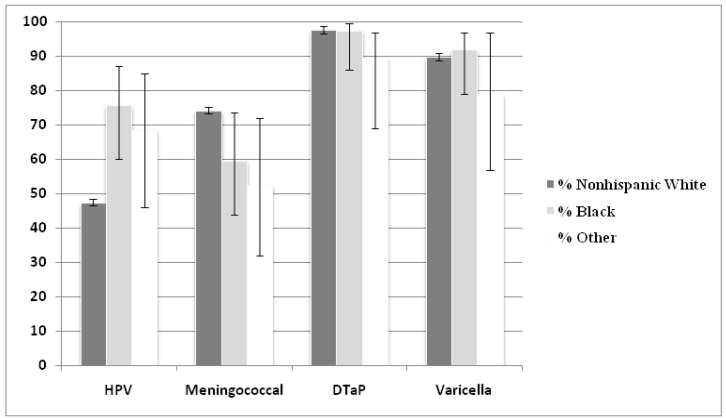
Vaccination rates of recommended adolescent vaccines by race.

After combining billing data with parental report, vaccine uptake rates were compared by race. Blacks and other races were significantly more likely than whites to have received at least one HPV vaccine injection (75.7% and 68.4% *vs.* 47.5%, respectively; *p* = 0.003). By comparison, whites were more likely to have received the meningococcal vaccine (74.3% *vs.* 59.5% and 52.6% respectively; *p* = 0.043), and rates were not significantly different for Tdap and varicella vaccines (Figure 2).

* Error bars represent 95% confidence interval.

**Figure 3:**
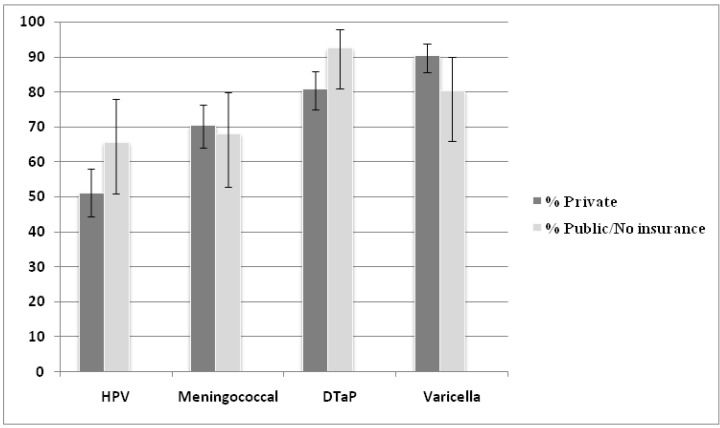
Vaccination rates of recommended adolescent vaccines by insurance status.

Further, rates of HPV vaccine uptake trended toward increased vaccination of children with public insurance or no insurance as compared to those with private insurance (66% *vs.* 51%; *p* = 0.06). Again, there were no significant differences for the other recommended childhood vaccines (Figure 3).

* Error bars represent 95% confidence interval.

In multivariable logistic regression, patients of black and other minority races were 4.9 and 4.2 times more likely to receive the HPV vaccine compared to their white counterparts. Recommendation by the child’s physician was determined by parents answering yes, no or unsure to the question “has your doctor recommended the HPV vaccination for your daughter?” Daughters of parents who answered yes to this question were twice as likely to initiate the HPV vaccine series. Similarly daughters of parents who disagreed or strongly disagreed with the statement “My daughter does not need the HPV vaccine” (a marker of perceived susceptibility) were also significantly more likely to be vaccinated. Safety concerns were the strongest attitude barrier to vaccination (Table 3). Insurance status was not independently associated with HPV vaccine uptake in the final model.

**Table 3:** Final regression model showing factors independently associated with initiating the HPV vaccine series.

	OR	95% CI	*p*-value
Black race *	4.9	1.8–13.6	0.0023
Other race *	4.2	1.1–16.6	0.042
**MD recommends**	2.1	0.97–4.4	0.06
**Perceived susceptibility**	1.7	1.1–2.6	0.014
**Safety concerns**	0.19	0.093–0.37	<0.0001

* As compared to nonhispanic white race.

### 3.2. Discussion

In this prospective cohort study, minority girls were more likely to receive the HPV vaccine compared to their nonhispanic white counterparts. In particular, race correlated more with vaccination initiation than insurance status, beliefs about safety and efficacy, recommendation by the pediatrician, and perceived susceptibility although these factors likely also played a role in the parents’ decision to have their child vaccinated. The CDC’s National Immunization Survey-Teen in 2011 documented a similar trend for higher vaccination rates among minorities [10]. However, the NIS-Teen data specifically looks at adolescents aged 13–17 and thus does not specifically address this issue of uptake at the time of the well child visit in younger adolescents. This study demonstrates that these differences may occur at the time of the initial adolescent visit and persistent throughout adolescence. Provider recommendation remains the strongest modifiable event to promote vaccination. However, recent data suggests that provider recommendation is lower for minority races [20]. Conversely, at least one study found that physicians with higher rates of minority patients had higher HPV vaccination rates, suggesting possible targeting of vaccine recommendation [21]. This study cannot provide any information about provider beliefs or attitudes although the strong association between MD recommendation and racial differences in uptake warrant further study in this area. Lastly, it is possible that perceived susceptibility could also play a role in increased uptake among minorities. Racial disparities in cervical cancer incidence and mortality are well documented and have been circulated widely in the lay public.

Among girls presenting to the doctor for a well-child visit in this study, privately insured patients were less likely to receive the HPV vaccine than girls with Medicaid or no insurance. This may be related to coverage and reimbursement issues which can be heterogeneous among private payers, possibly resulting in more out-of-pocket costs for parents. By comparison, all children who qualify for Medicaid and/or are underinsured for vaccinations and meet certain financial cutoffs are covered by the Federal Vaccines for Children program for all vaccines recommended by the CDC, including the HPV vaccine. The NIS-Teen survey also noted a difference in vaccination by socioeconomic status with 46.4% of girls below the poverty level initiating vaccination compared to 35.8% of girls at or above the poverty level choosing to be vaccinated [10]. It is possible in our study, that any role insurance status plays is mediated by the difference in race among payer groups. Future studies should assess this in more detail in the questionnaire.

This study evaluated HPV vaccination given uniform access to care in a single cohort of girls being seen for well child checks in the same health care system. This is a strength of the study as it controls for regional differences in provider counseling and issues in access to patient care. This research utilized prospective data collection and objective billing data for confirmation of vaccination status allowing for the most accurate determination of vaccination. In addition, utilization of a professional survey group for interviews, validated questionnaire, and a piloted study technique allows for high quality subjective data. This study is limited by its small sample size and evaluation of only a single health care delivery system. For the follow-up questionnaire, we had a relatively low response rate but this is comparable to similar telephone surveys conducted on HPV vaccination [11]. In regards to nonresponder bias, we found that patients with public insurance were less likely to participate in the questionnaire. This may limit our abilities to fully characterize this group’s higher vaccination rates. Further, our research by the nature of cohort selection cannot take into account vaccination barriers related to access to care or determine causation.

This study documents HPV vaccination rates, attitudes and barriers prior to initiation of a statewide mandate requiring girls in Virginia to receive the HPV vaccine prior to their 11th birthday for school admission and begins to identify further areas that need elucidation. These data will serve as the baseline for a comparison study of HPV vaccination rates and counseling 5 years after enactment of the mandate. This study is uniquely positioned to address this question given the objective data capture through the billing database combined with a validated parental survey and uniform access to care. Further, documented differences by race and insurance status seen with HPV vaccination but not with other adolescent immunizations will serve as the baseline for post-mandate comparisons. The follow-up study is slated to be conducted starting in August 2013. These data may have implications beyond a single state given similar trends in differential rates of uptake by race seen at a national level.

## 4. Conclusions

In this study, we found that parents report high rates of understanding that the HPV vaccine is safe, effective, and recommended for adolescent girls. Privately insured patients had lower vaccination rates compared to those with public or no insurance. Racial differences exist with minority girls having higher HPV vaccination rates than their white counterparts. Future studies will be necessary to evaluate whether the verbal opt-out mandate in Virginia will have any effect on these racial differences.

## Supplementary Material

**Table S1:** HPV vaccine uptake by insurance status and race: supplemental data for Table 2.

		HPV vaccine uptake	%	95% CI
**Insurance status**	**Private **	130/292	44.5%	38.9–50.3
	**Public **	103/175	58.9%	51.5–65.9
	**Self-pay **	11/22	50%	31–69
	**Unknown **	21/61	34%	24–47
**Race**	**Nonhispanic white **	172/396	43.4%	38.6–48.3
	**Black **	70/115	60.9%	51.7–69.3
	**Other **	23/39	59%	43–73
**Total**		265/550	48.2%	44–52.4

**Table S2:** Supplemental data for Figure 2.

Vaccine	White * n = 179	%	95% CI	Black * N = 37	%	95% CI	Other * N = 19	%	95% CI
HPV	85	47.5	40%–55%	28	75.7	60%–87%	13	68.4	46%–85%
Meningococcal	133	74.3	67%–80%	22	59.5	44%–74%	10	52.6	32%–72%
DTaP	175	97.7	94%–99%	36	97.3	86%–99%	17	89.5	69%–97%
Varicella	161	89.9	85%–94%	34	91.9	79%–97%	15	79	57%–91%

* Data missing for 4 patients of white race, 1 patient of black race, and 8 patients of other race.

**Table S3:** Supplemental data for Figure 3.

Vaccine	Private n = 201	%	95% CI	Public/no insurance n = 41	%	95% CI
HPV	103	51.2	44.4–58.1	27	65.8	51–78
Meningococcal	142	70.7	64–76.5	28	68.3	53–80
DTaP	163	81	75.1–85.9	38	92.7	81–98
Varicella	182	90.6	85.7–93.9	33	80.5	66–90

**Table S4:** Insurance status by race.

Insurance status	Nonhispanic white n = 396	Black n = 115	Other n = 39
Private	235 (59.3%)	38 (33%)	19 (48.7%)
Public	101 (25.5%)	61 (53%)	11 (28.2%)
Self pay	13 (3.3%)	9 (7.8%)	2 (5.1%)
Unknown	47 (11.9%)	7 96.1%)	7 (17.9%)
